# Metabolic pathways enriched according to ERG status are associated with biochemical recurrence in Hispanic/Latino patients with prostate cancer

**DOI:** 10.1002/cam4.5301

**Published:** 2022-11-03

**Authors:** Natalia L. Acosta‐Vega, Rodolfo Varela, Jorge Andrés Mesa, Jone Garai, Melody C. Baddoo, Alberto Gómez‐Gutiérrez, Silvia J. Serrano‐Gómez, Marcela Nuñez Lemus, Martha Lucía Serrano, Jovanny Zabaleta, Alba L. Combita, María Carolina Sanabria‐Salas

**Affiliations:** ^1^ Grupo de Investigación en Biología del Cáncer Instituto Nacional de Cancerología de Colombia Bogotá DC Colombia; ^2^ Programa de doctorado en Ciencias Biológicas Pontificia Universidad Javeriana Bogotá DC Colombia; ^3^ Departamento de Urología Instituto Nacional de Cancerología de Colombia Bogotá DC Colombia; ^4^ Departamento de Patología Oncológica Instituto Nacional de Cancerología de Colombia Bogotá DC Colombia; ^5^ Stanley S. Scott Cancer Center Louisiana State University Health Sciences Center New Orleans Louisiana USA; ^6^ Tulane University School of Medicine New Orleans Louisiana USA; ^7^ Instituto de Genética Humana, Facultad de Medicina Pontificia Universidad Javeriana Bogotá DC Colombia; ^8^ Grupo de Apoyo y Seguimiento para la Investigación Instituto Nacional de Cancerología de Colombia Bogotá DC Colombia; ^9^ Departamento de Química, Facultad de Ciencias Universidad Nacional de Colombia Bogotá DC Colombia; ^10^ Department of Interdisciplinary Oncology School of Medicine, Louisiana State University Health Sciences Center New Orleans Louisiana USA; ^11^ Departamento de Microbiología, Facultad de Medicina Universidad Nacional de Colombia Bogotá DC Colombia

**Keywords:** biochemical recurrence, differentially expressed genes, *ERG* subtypes, prostatic neoplasms, signaling pathways

## Abstract

**Background:**

The role of *ERG*‐status molecular subtyping in prognosis of prostate cancer (PCa) is still under debate. In this study, we identified differentially expressed genes (DEGs) according to *ERG*‐status to explore their enriched pathways and implications in prognosis in Hispanic/Latino PCa patients.

**Methods:**

RNA from 78 Hispanic PCa tissues from radical prostatectomies (RP) were used for RNA‐sequencing. *ERG*_*high*_/*ERG*_*low*_ tumor groups were determined based on the 1.5‐fold change median expression in non‐tumor samples. DEGs with a False Discovery Rate (FDR) < 0.01 and a fold change >2 were identified between *ERG*_*high*_ and *ERG*_*low*_ tumors and submitted to enrichment analysis in MetaCore. Survival and association analyses were performed to evaluate biochemical recurrence (BCR)‐free survival.

**Results:**

The identification of 150 DEGs between *ERG*_*high*_ and *ERG*_*low*_ tumors revealed clustering of most of the non‐BCR cases (60%) into de *ERG*_*high*_ group and most of the BCR cases (60.8%) in *ERG*_*low*_ group. Kaplan–Meier survival curves showed a worst BCR‐free survival for *ERG*_*low*_ patients, and a significant reduced risk of BCR was observed for *ERG*_*high*_ cases (OR = 0.29 (95%CI, 0.10–0.8)). Enrichment pathway analysis identified metabolic‐related pathways, such as the renin‐angiotensin system and angiotensin maturation system, the linoleic acid metabolism, and polyamines metabolism in these *ERG* groups.

**Conclusions:**

*ERG*_*low*_ tumor cases were associated with poor BCR‐free survival in our Hispanic/Latino patients, with metabolism‐related pathways altered in the BCR progression.

**Impact:**

Our findings suggest the need to dissect the role of diet, metabolism, and lifestyle as risk factors for more aggressive PCa subtypes.

## Graphical abstract

Differentially expressed genes (DEGs) between *ERG*_*high*_ and *ERG*_*low*_ tumors revealed clustering of most of the non‐BCR cases into de *ERG*_*high*_ group and most of the BCR cases in *ERG*_*low*_ group. Subsequent analyses confirmed an association between ERG status with BCR, showing a worst BCR‐free survival for *ERG*_*low*_ patients compared to the *ERG*_*high*_ group. Enrichment pathway analysis of the 150 DEGs identified an important participation of metabolic‐related pathways in the BCR progression.

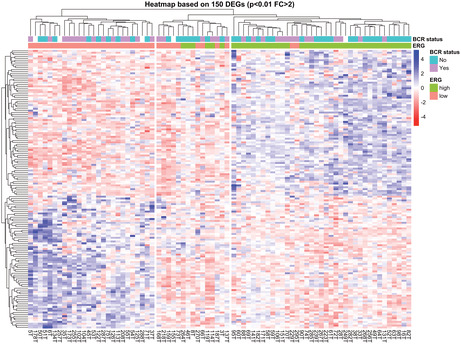

## 1 INTRODUCTION

Prostate cancer (PCa) is the second most common cancer and the fifth leading cause of death from cancer in men worldwide.^1^ In Colombia, PCa is the most common cancer in men with estimated age‐standardized incidence rates of 49.8 cases per 100,000 inhabitants and second highest mortality rates with 12–12.6 per 100,000 inhabitants.^1^, ^2^

The understanding of PCa molecular alterations has increased with the definition of molecular subtypes and the identification of prognostic gene‐expression signatures.^3^ The establishment of subtypes began with the identification of the fusion of genes *ERG* and *TMPRSS2* as a common somatic alteration in PCa. *TMPRSS2:ERG* (T2E) gene fusion results in overexpression of *ERG*, a known oncogene and member of the ETS transcription factor family.^4^, ^5^ Around 50% of Caucasians PCa patients harbor T2E‐positive tumors, but lower frequencies have been reported in men of African or Asian ethnicities.^6^ Later, it was established that negative *ERG* tumor status was associated with poorer BCR‐free survival in Caucasian, but no relation was found in African–American patients.^7^ Moreover, there are currently several publications that have shown an association between high expression of *ERG* with good prognosis,^7^, ^8^, ^9^ whereas others report an inverse association.^10^, ^11^ A meta‐analysis including 48 studies showed no evidence of an association with recurrence‐free or disease‐specific survival,^12^ though authors conclude *ERG* status might allow patient stratification for different management strategies.^13^, ^14^ Therefore, there is still conflicting evidence as to whether the T2E fusion and/or the level of *ERG* expression have prognostic implications.^15^ Also, the dissimilarities in the frequency of the fusion and prognosis may be given by differences in the genetic structure of the ethnic groups.

Given that the T2E gene fusion is an early event in PCa, fusion‐positive tumors are believed to represent a distinct molecular subtype of PCa involving activation of specific oncogenic pathways,^16^, ^17^ as well as different metabolic profiles compared with the T2E fusion‐negative tumors.^18^, ^19^ Therefore, in this study, we aimed to explore molecular differences associated to progression in *ERG*_*high*_ and *ERG*_*low*_ PCa tumors through a differential expression analysis and enrichment pathway analysis in Hispanic/Latino patients with localized/regionally advanced PCa.

## 2 MATERIAL AND METHODS

### 2.1 Patients and sample collection

Localized/regionally advanced PCa patients diagnosed at Instituto Nacional de Cancerología (INC) in Colombia between 2007 and 2011 were included. Samples were obtained from FFPE (Formalin Fixed Paraffin Embedded) tissues from radical prostatectomies (RP). This protocol was approved by the Research Ethics Board at the INC and was designated as an exempt study for informed consent.

One hundred and one (n = 101) suitable cases were identified through histologic review by an expert pathologist. All tumor samples with Gleason pattern over 3 + 3 and high‐density areas of tumor cells ≥65%, as well as non‐tumor regions, were selected. Section cores were extracted for each type of tissue. From each RP, only the focus with the highest Gleason pattern from each patient was used in this study. Clinical information was obtained from INC databases. BCR was defined as the elevation of serum PSA levels over 0.2 ng/mL on two successive measurements, as previously established^20^; for this work, BCR was defined within the 5 years of follow‐up after RP surgery.

### 2.2 RNA extraction

Total RNA was extracted using AllPrep DNA/RNA FFPE kit® (Qiagen, Hilden, Germany) following the manufacturer's recommendations. RNA quantity and quality were determined with Nanodrop 2000 Spectrophotometer® (ThermoFisher Scientific, Wilmington, USA) and the Agilent RNA 6000 Nano kit® (Agilent Technologies, Santa Clara, CA), respectively. All samples were suitable for library preparation.

### 2.3 RNA‐Seq library preparation and sequencing

For library preparation, 1 μg of total RNA and the TruSeq Stranded Total RNA Library Prep kit® with Ribo‐Zero Human/Mouse/Rat (Illumina, Inc., San Diego, CA, USA) were used. Fragmentation step was omitted in most of the samples due to sample quality, while in five samples, it was done according to recommendations from Illumina®. Validation of libraries was performed in the Agilent 2100 Bioanalyzer® system and then normalization to 10 nM was done with the Qubit dsDNA HS Assay kit® (ThermoFisher Scientific, Wilmington, USA), before cluster generation. Sequencing was performed in 12‐pooled samples at 1 × 75 bp with single‐end strategy in a NextSeq 500® (Illumina Inc., San Diego, CA, USA), with no sequencing results in six tumor samples.

### 2.4 RNA‐seq data analysis

Reads were checked for quality control (QC) using FASTQC and then aligned to human ribosomal RNA using STAR® v.2.5.2.^21^ Fifteen tumor samples had rRNA content higher than 70% and were excluded. Unmapped reads were used to map to human reference Homo_sapiens.GRCh38.78 (Ensembl) using RSEM® v1.2.31^22^ to generate the read counts and expression calculations. Filtering according to ENSEMBL protein coding genes was done. Filtering of outlier samples through principal component analysis (PCA) excluded 2 tumor samples remaining 78 tumor samples. Genes with median counts of zero in all samples were also filtered out and PCA was used to check batch effects to correct in further analyses.

### 2.5 Determining *ERG*_*high*_ and *ERG*_*low*_

To determine tumor cases with *ERG*_*high*_ and *ERG*_*low*_, we calculated a 1.5‐fold change over the *ERG* median expression value in non‐tumor tissues as the cut‐off point. Tumor cases above the defined value were classified as *ERG*_*high*_, otherwise tumors were categorized as *ERG*_*low*_.

### 2.6 Ancestry estimation

DNA from adjacent non‐tumoral FFPE tissue from 101 cases was extracted using AllPrep DNA/RNA FFPE kit® (Qiagen, Hilden, Germany) following the manufacturer's recommendations. Samples were sent to the University of Minnesota Genomics Center for genotyping of 106 autosomal Ancestry‐Informative Markers (AIMs),^23^ in a Sequenom iPLEX® Genotyping Platform. Single nucleotide polymorphisms (SNPs) with call rate lower than 90% were removed, leaving 101 for ancestry estimation; similarly, 25 samples with a call rate lower than 85% were excluded, remaining 76 cases. The concordance score for genotyping was 97.4% between 22 duplicated samples. Additionally, all AIMs were in Hardy–Weinberg equilibrium. These analyses were done in PLINK® v1.90b4.1 64‐bit. Finally, proportions of European, African, and Indigenous American genetic ancestry were estimated for each case with the ADMIXTURE® software V1.3.0 under an admixture model. To perform a supervised analysis, three parental reference populations were included: European (42 individuals from Coriell's North American Caucasian panel), African (37 non‐admixed Africans living in United Kingdom and South Carolina—USA), and Indigenous Central American populations (15 Mayan and 15 Nahuas).^23^

### 2.7 Differential gene expression analysis

Filtered raw counts from 78 samples were used as input data for analysis in DESeq2® package v1.20.0.^24^ Pre‐filtering to include transcripts with at least 1 read count in more than 80% of the samples to count data was applied. Comparisons between *ERG*_*high*_ and *ERG*_*low*_ tumors were made to identify the differentially expressed genes (DEGs). Estimated genetic ancestry was also included as a variable to determine the effect of ancestry in the differential gene expression analysis, we only included Indigenous and European ancestries proportion since we found a low representation of African ancestry in our cases (median percentage of 5%). All the comparisons included batch correction following recommendations documented for the package. Genes with False Discovery Rate (FDR) less than 0.01 and fold change over 2 were selected as DEGs. Unsupervised clustering was done with normalized expression data of DEGs by using *Pheatmap*® package v1.0.10.

### 2.8 *ERG* expression dataset from GEO repositories

The GEO dataset GSE70770^25^ was used to confirm the association of *ERG* expression with BCR. *ERG* was categorized as high and low based on the median normalized counts of expression to determine the implication of *ERG* with BCR‐free survival through Kaplan–Meier survival curve and log‐rank test.

### 2.9 Statistical analysis

For clinical‐pathological characteristics, continuous variables were analyzed applying analysis of variance test (ANOVA) and Kruskal–Wallis for multiple comparisons and Student's T test and Wilcoxon rank‐sum test for comparisons between two groups. Categorical variables were analyzed with *X* ^*2*^ test and Fisher's exact test. The assumption of normally distributed data was tested by Shapiro–Wilk test. Principal component analysis with RNA‐seq data was done by using singular value decomposition (SVD) on the Log2 transformed counts. Kaplan–Meier survival curves and log‐rank test were done with R Survival® and Survminer® packages for associations between *ERG* status with BCR‐free survival. *p*‐value <0.05 was considered statistically significant. Univariate logistic regressions with estimated ORs and 95% confidence intervals (CI) were assessed for associations between ERG and clinical‐pathological variables with BCR. Variables with statistically significant *p*‐values <0.1 were included in a multivariate model for logistic regression. All the assumptions were verified, and to assess model fit we used goodness of fit measurements. Statistical analyses were done in Rstudio® v1.1.463.

## 3 RESULTS

### 3.1 Patient clinicopathological characteristics

The 78 sequenced tumor cases are described in Table 1. BCR information was available for 73 cases from which 34 (46.6%) presented BCR within a median time of 16.59 months (range 2.1–55.07 months) in the 5‐years of follow‐up after the RP surgery. *ERG* expression was low in 47.4% of cases and high in 52.6% (Table 1).

**TABLE 1:** Clinical and pathological characteristics of analyzed patients and distribution of clinical and pathological characteristics stratified by *ERG* groups

Characteristics	N = 78	*ERG*_*low*_ n = 37	*ERG*_*high*_ n = 41	*p*
Age ‐ years (median, range)	65 (32–73)	66 (32–73)	64 (42–73)	0.616
Age ‐ years (%)				
<50	7 (9.0)	2 (5.4)	5 (12.2)	0.681
50–60	12 (15.4)	7 (18.9)	5 (12.2)	
60–70	46 (59.0)	22 (59.5)	24 (58.5)	
>70	13 (16.7)	6 (16.2)	7 (17.1)	
BMI (median [range])	26.57 (17.28–36)	26.75 (17.28–35.19)	25.47 (17.71–36)	0.216
Ancestry (median, range)				
European ancestry	0.57 (0.19–0.81)	0.54 (0.19–0.81)	0.58 (0.23–0.78)	0.756
Indigenous ancestry	0.38 (0.01–0.66)	0.39 (0.05–0.66)	0.37 (0.01–0.65)	0.713
African ancestry	0.05 (0.00–0.58)	0.06 (0.00–0.56)	0.04 (0.00–0.58)	0.76
Pre‐operative characteristic				
Preoperative PSA (median, range)	9.41 (2.94–45.21)	9.40 (2.94–45.21)	9.41 (3.7–44)	0.579
Clinical stage (%)				
I	24 (30.8)	10 (27.0)	14 (34.1)	0.545
II	53 (67.9)	26 (70.3)	27 (65.9)	
IV	1 (1.3)	1 (2.7)	0 (0.0)	
Gleason Grade Group at biopsy (%)				
GG1	41 (56.9)	13 (40.6)	28 (70.0)	0.063
GG2	15 (20.8)	8 (25.0)	7 (17.5)	
GG3	11 (15.3)	7 (21.9)	4 (10.0)	
GG4 and GG5	5 (6.9)	4 (12.5)	1 (2.5)	
D'Amico risk groups (%)				
LR	21 (26.9)	9 (24.3)	12 (29.3)	0.806
IR	36 (46.2)	17 (45.9)	19 (46.3)	
HR	21 (26.9)	11 (29.7)	10 (24.4)	
Post‐operative characteristic				
% tumor in RP (median, range)	18.50 (1–90)	21 (1–90)	16 (1–75)	0.357
Gleason Grade Group at PR (%)				
GG1	22 (28.2)	5 (13.5)	17 (41.4)	**0.036**
GG2	24 (30.8)	12 (32.4)	12 (29.3)	
GG3	18 (23.1)	11 (29.8)	7 (17.1)	
GG4 and GG5	14 (17.9)	9 (24.3)	5 (12.2)	
Pathological stage (%)				
T1/T2	41 (52.6)	18 (48.6)	23 (56.1)	0.65
T3	37 (47.4)	19 (51.4)	18 (43.9)	
Lymphovascular Invasion in RP (%)				
No	55 (85.9)	25 (80.6)	30 (90.9)	0.296
Yes	9 (14.1)	6 (19.4)	3 (9.1)	
Perineural invasion in RP (%)				
No	12 (16.4)	7 (19.4)	5 (13.5)	0.543
Yes	61 (83.6)	29 (80.6)	32 (86.5)	
Extracapsular extension in RP (%)				
No	38 (50.0)	18 (50.0)	20 (50.0)	1
Yes	38 (50.0)	18 (50.0)	20 (50.0)	
Lymph node compromise (%)				
No	67 (85.9)	31 (83.8)	36 (87.8)	0.748
Yes	11 (14.1)	6 (16.2)	5 (12.2)	
Follow‐up characteristic				
Additional treatment (%)^a^				
No	55 (70.5)	22 (59.5)	33 (80.5)	0.05
ADT	23 (29.5)	15 (40.5)	8 (19.5)	
BCR (%)				
No	39 (53.4)	12 (36.4)	27 (67.5)	**0.01**
Yes	34 (46.6)	21 (63.6)	13 (32.5)	
PSA at BCR (median [range])	0.27 (0.20–3.54)	0.32 (0.20–3.54)	0.26 (0.21–0.41)	0.232
Time to BCR – months (median [range])	16.59 (2.10–55.07)	22.2 (4–55.1)	8.1 (2.1–42.1)	0.074
Time of follow‐up ‐ months (median [range])	67.60 (3.37–112.63)	66.9 (4.9–111.4)	69.4 (3.4–112.6)	0.806

Abbreviations: ADT, androgen deprivation therapy; BMI, body mass index; PSA, prostate‐specific antigen; RP, radical prostatectomy;.

^a^ Additional treatment: received after the radical prostatectomy treatments and after the biochemical recurrence.

Clinicopathological characteristics compared between *ERG* groups showed that *ERG*_*low*_ group have higher frequency of higher Gleason Grades at RP (54.1% accounting for GG3‐GG4/GG5) compared with *ERG*_*high*_ (29.3% for GG3‐GG4/GG5) (Table 1) while *ERG*_*high*_ group was enriched in lower Gleason Grades (70.7% for GG1‐GG2; *p* = 0.036) (Table 1). Of notice, BCR was also statistically significant different between the two groups, with 63.6% of BCR cases in *ERG*_*low*_ and only 32.5% of BCR cases in *ERG*_*high*_ group (Table 1), which suggest an association of *ERG*‐status with prognosis for these localized and regionally advanced PCa patients.

### 3.2 Differentially expressed genes between ERG groups

**FIGURE 1:**
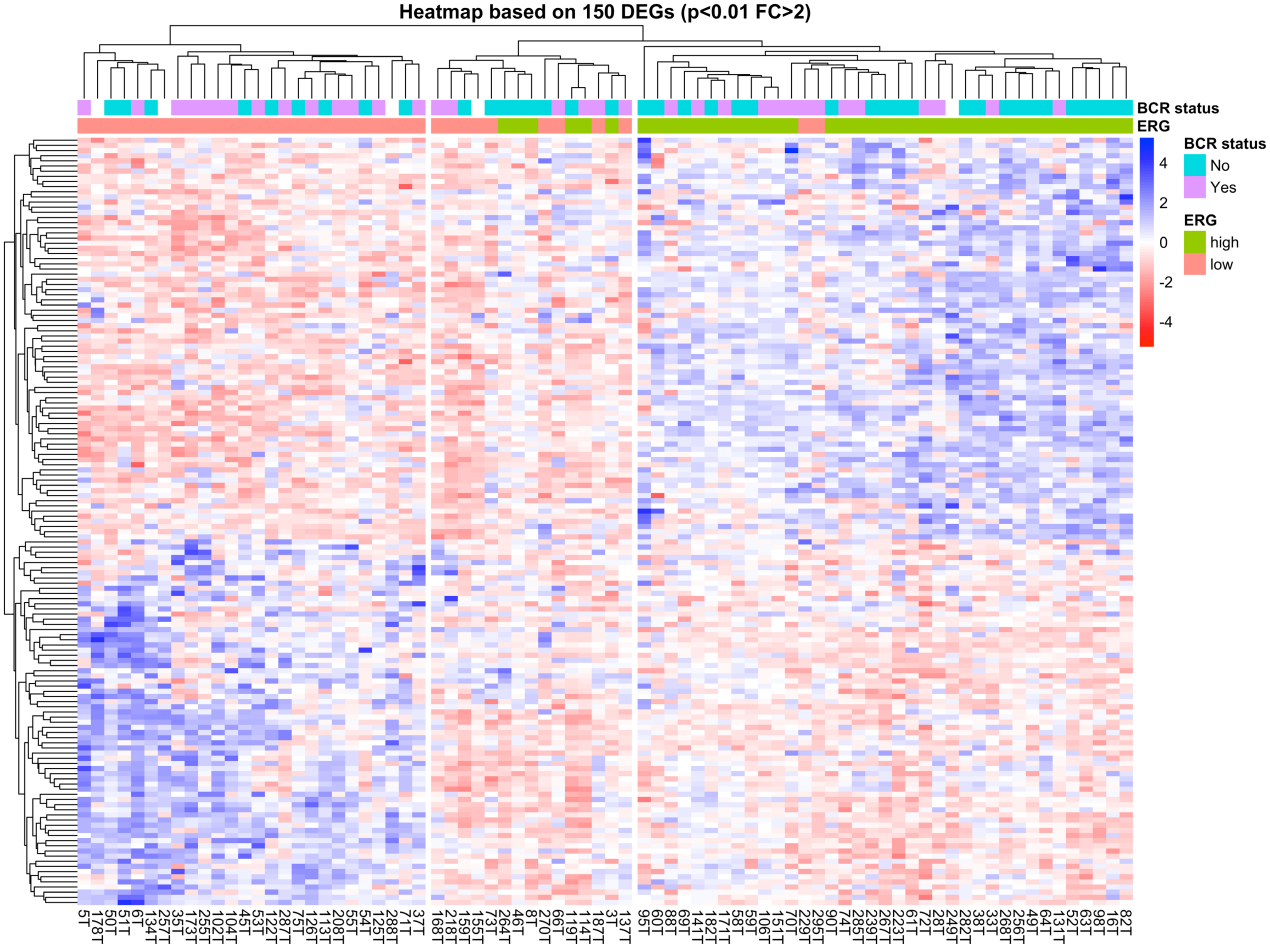
Heatmap for the 150 DEGs. Unsupervised hierarchical clustering analysis for 150 DEGs in 78 tumor samples from PCa patients. DEGs were obtained from comparison between *ERG*_*high*_ and *ERG*_*low*_ groups (FDR <0.01, fold change >2). Normalized counts of expression were scaled, and expression values for each gene were color labeled (blue to red). Patients are represented in columns and genes in rows. The separation in clusters shows the 15 samples forming a sub‐cluster within the ERGhigh tumors.

DESeq2 results between the two *ERG* tumor groups showed 532 DEGs, including 284 overexpressed and 248 with low expression in the group of *ERG*_*high*_ compared to *ERG*_*low*_ tumors using an FDR <0.01 (Figure S1). Setting a fold change over 2, 150 DEGs remained which, based on their expression, were able to separate most *ERG*_*high*_ from *ERG*_*low*_ tumors into two clusters through a hierarchical clustering analysis (Figure 1). Interestingly, as it is shown in the heatmap, most of the PCa cases with BCR were grouped within the *ERG*_*low*_ cluster (14/23, 60.8%), while most of the non‐BCR cases were grouped within the *ERG*_*high*_ tumors (30/50, 60%). These results are in line with the suggested associations that we found in the clinicopathological analysis in which most of the BCR cases were in the *ERG*_*low*_ group and most non‐BCR in the *ERG*_*high*_ group (Table 1).

In addition, it is noteworthy that a small group of 15 samples form a sub‐cluster within the *ERG*_*high*_ tumors (Figure 1). These 15 samples, located in the sub‐cluster to the left under the *ERG_high_* cluster in the dendrogram, differ in the gene expression pattern (see genes in rows) compared with the whole cluster for *ERG*_*high*_, especially in the first panel of genes. Of notice, these cases are enriched in *ERG*_*low*_ cases (9 out of 15) (Figure 1). Clinicopathological characteristics between this group compared with the *ERG*_*high*_ and *ERG*_*low*_ clusters showed significant statistical differences within Gleason GG at RP, with 33.3% in each of the Gleason groups GG3 and GG4/GG5, for a total of 66.6% of cases associated with the higher Gleason groups (GG3 and GG4/GG5) (Table S1). This contrasts with findings in *ERG*_*low*_ and *ERG*_*high*_ groups presenting 46.1% and 27% of cases, respectively, associated with Gleason groups GG3 and GG4/GG5. However, we did not find association with BCR (Table S1).

### 3.3 Effect of genetic ancestry in the identification of DEGs

We wanted to determine whether genetic ancestry modulates the expression of genes associated to the *ERG* expression. We included the Indigenous and European genetic ancestries in the analysis of differential expression in DESeq2 comparing the *ERG* groups. No DEGs were found other than the obtained without including this variable, suggesting that the European and Indigenous ancestries, as analyzed here, do not modify differentially expressed genes found between *ERG* groups in our patients. We included only Indigenous and European ancestries since they sum for the major genetic component in the population included in this study.

### 3.4 BCR‐free survival analysis according to *ERG* groups

**FIGURE 2:**
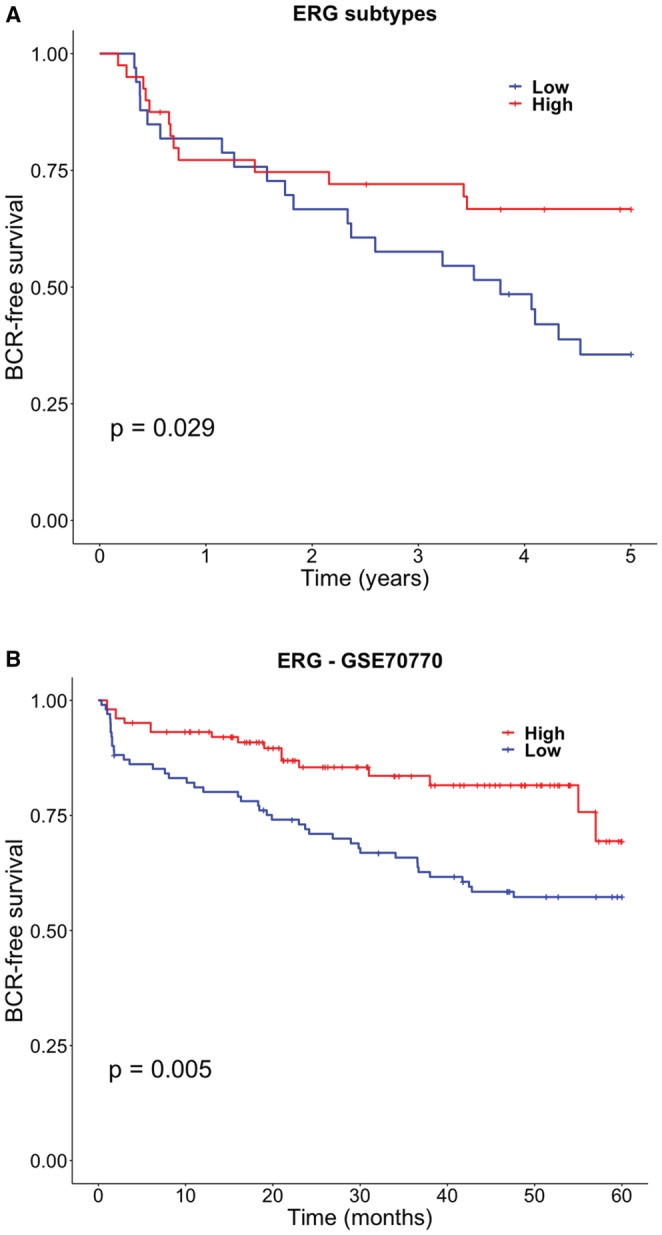
Survival curves for *ERG* tumor groups. (A) Kaplan–Meier curve for BCR‐free survival in years for 73 PCa patients with ERGlow (blue) and *ERG_high_* (red) expression. (B) Kaplan–Meier curve for BCR‐free survival in the GSE70770 dataset by ERGlow (blue) and *ERG_high_* (red) expression. The comparison method for the survival curves was Log‐rank test.

To evaluate the impact of the *ERG*‐status on prognosis, we assessed the association between both groups with BCR‐free survival by Kaplan–Meier analysis. It showed that *ERG*_*low*_ group was correlated with worse BCR‐free survival within 5 years of RP (log‐rank test *p* = 0.029) (Figure 2A) and univariate logistic regressions confirmed the association between risk of BCR with *ERG*_*high*_ group as a protector factor (OR = 0.28; 95%CI, 0.10–0.71; *p* = 0.009) (Table 2).

**TABLE 2:** Associations between clinical‐pathological variables and *ERG* groups with BCR through univariate and multivariate logistic regression analyses

Clinical‐pathological variables	Univariate logistic regression			Multivariate logistic regression		
	OR	95% CI	*p* value	OR	95% CI	*p* value
Age ‐ years	0.97	0.92–1.03	0.380			
BMI	0.99	0.87–1.14	0.934			
European ancestry	0.47	0.01–24.15	0.707			
Indigenous ancestry	0.85	0.02–46.07	0.934			
African ancestry	2.51	0.05–165.01	0.646			
Pre‐operative characteristic						
Preoperative PSA	0.98	0.93–1.04	0.542			
Clinical stage						
I	Ref.					
II	1.81	0.67–5.08	0.245			
Gleason Grade Group at biopsy						
GG1	Ref.					
GG2	1.16	0.32–4.12	0.817			
GG3	1.35	0.33–5.60	0.670			
GG4 and GG5	4.06	0.47–6.08	0.242			
Post‐operative characteristic						
Gleason Grade Group at RP						
GG1	Ref.			Ref.		
GG2	3.73	1.12–13.69	**0.037**	2.95	0.83–11.25	0.101
GG3	2.37	0.63–9.43	0.205	1.62	0.39–6.88	0.504
GG4 and GG5	4.00	0.86–21.05	0.084	2.81	0.55–15.71	0.219
Pathological stage						
T1/2	Ref.					
T3	2.26	0.89–5.91	**0.089**			
% tumor in RP	1.02	0.99–1.05	0.139			
Lymphovascular Invasion in RP (%)						
No	Ref.					
Yes	0.94	0.17–4.65	0.937			
Perineural invasion in RP						
No	Ref.					
Yes	3.22	0.86–15.68	0.103			
Extracapsular extension in RP (%)						
No	Ref.					
Yes	2.08	0.81–5.47	0.130			
Lymph node compromise (%)						
No	Ref.					
Yes	0.91	0.21–3.73	0.891			
Index of dominant tumor nodule	1.58	0.69–3.82	0.286			
D'Amico risk groups (%)						
LR	Ref.					
IR	1.11	0.37–3.41	0.851			
HR	1.48	0.43–5.28	0.537			
PSA after RP	0.90	0.54–1.05	0.506			
*ERG* groups						
Low	Ref.			Ref.		
High	0.28	0.10–0.71	**0.009**	0.32	0.11–0.88	**0.029**

Abbreviations: BMI, body mass index; PSA, prostate‐specific antigen; RP, radical prostatectomy.

We also determined the associations of clinical‐pathological variables with the risk of BCR. In the univariate logistic regression, only Gleason GG2 at RP was associated (OR = 3.73; 95%CI 1.12–13.69; *p* = 0.037) (Table 2). For the multivariate logistic regression model, we included Gleason GGs at RP and *ERG* groups, since they were significant in the univariate analyses. Only *ERG* groups maintained significant, with an OR of 0.29 (95%CI, 0.10–0.8; *p* = 0.020) for *ERG*_*high*_ group (Table 2).

Next, we used the GSE70770 dataset (n = 203) to validate in an independent set whether *ERG* expression is associated with BCR (BCR, n = 59). Cases were divided according to the normalized *ERG* expression into *ERG*_*high*_ and *ERG*_*low*_. These groups analyzed by Kaplan–Meier showed statistically significant differences in BCR‐free survival confirming *ERG*_*low*_ as the group with shorter BCR‐free survival compared with *ERG*_*high*_ group (*p* = 0.005) (Figure 2B). Cox proportional hazard model regression also showed higher BCR risk for *ERG*_*low*_ group (Hazard ratio = 2.2; 95%CI, 1.3–3.9; *p* = 0.004).

### 3.5 Signaling pathway analysis

**FIGURE 3:**
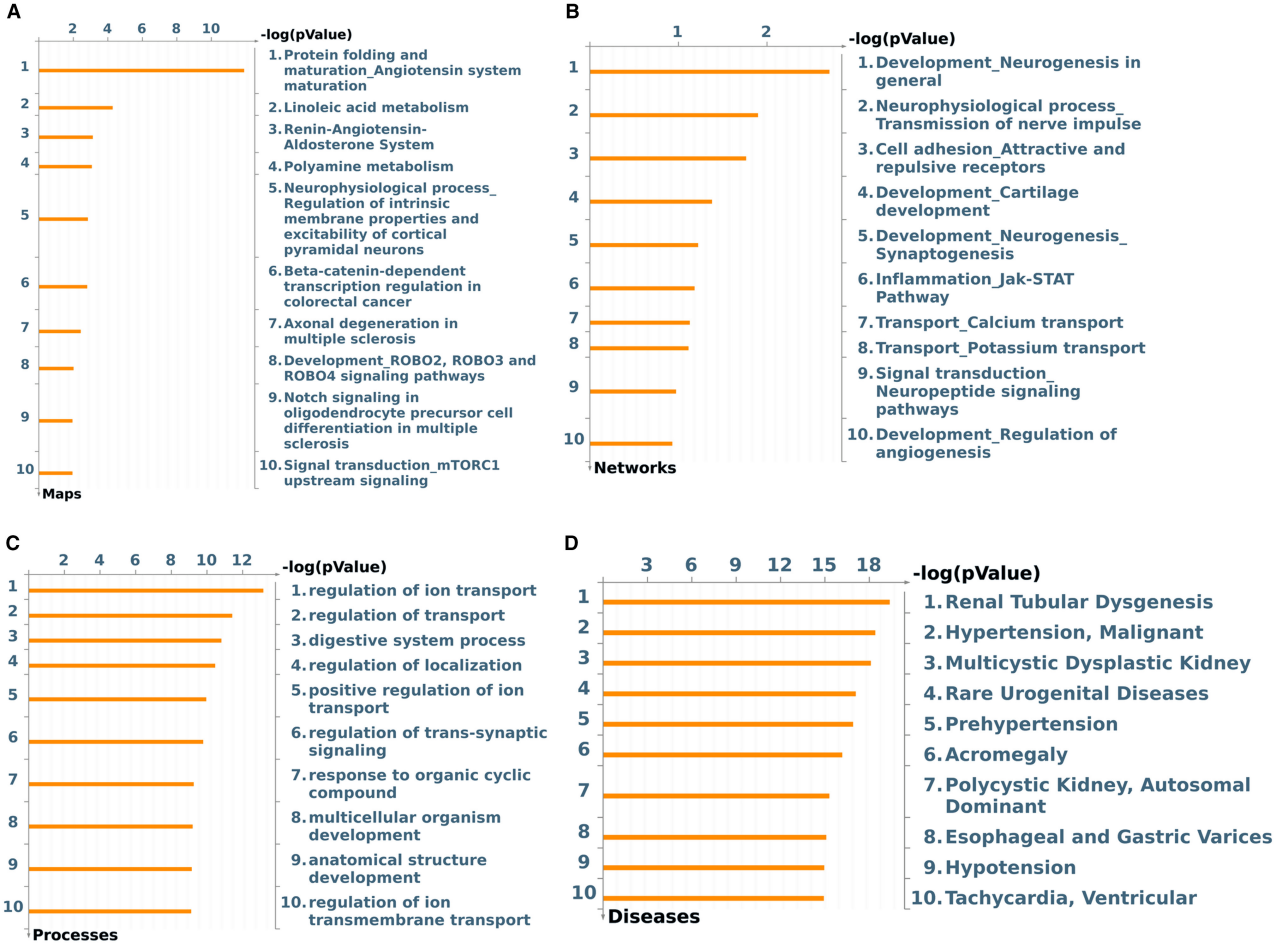
Enrichment analysis for 150 DEGs obtained for ERGhigh vs. ERGlow. (A) Pathway maps. (B) Networks. (C) Processes. (D) Diseases. DEGs were selected with and FDR <0.01 and fold change >2 and submitted to MetaCore for analysis.

Given that DEGs were able to separate most of the samples between *ERG*_*high*_ and *ERG*_*low*_ tumor groups, we explored how these DEGs participate in signaling pathways and processes that could contribute to the prognosis of the disease. Among the most significant pathways maps identified, as is shown in Figure 3A, we found those related with Angiotensin, such as are Protein folding and maturation_Angiotensin system maturation and Renin‐Angiotensin‐Aldosterone System; pathways maps related with metabolism, such as linoleic acid metabolism and polyamine metabolism. Other pathways identified were Beta‐catenin‐dependent transcription regulation in colorectal cancer; Development_ROBO2, ROBO3, and ROBO4 signaling pathways, Notch signaling in oligodendrocyte precursor cell differentiation in multiple sclerosis and Signal transduction_mTORC1 upstream signaling. The DEGs that participate in each of these pathways and direction of expression in *ERG*_*high*_ and *ERG*_*low*_ tumor groups are listed in Table 3.

**TABLE 3:** DEGs found associated with the most significantly enriched pathways. Direction of the expression identified by DESeq2 analysis is represented by *ERG*‐status according to the values of the Log2 fold change

Pathway Map ‐ DEGs	Log 2 Fold Change	*ERG*_*high*_	*ERG*_*low*_
Maturation_Angiotensin system maturation			
*ANT* ‐ Angiotensinogen	1.58	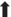	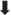
*ANPEP* ‐ Alanyl aminopeptidase, membrane	−2.97	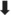	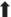
*MME* ‐ Neprilysin	−139.83	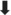	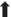
**Linoleic acid metabolism**			
*CYP2J2* ‐ Cytochrome P450 family 2 subfamily J member 2	−1.24	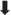	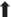
*ALOX15* ‐ Arachidonate 15‐lipoxygenase	1.31	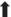	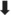
*FADS2* ‐ Fatty acid desaturase 2	−1.004	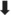	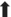
*ALOX15B* ‐ Arachidonate 15‐lipoxygenase type B	−2.97	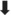	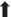
**Renin‐Angiotensin‐Aldosterone System**			
*ANT* ‐ Angiotensinogen	1.58	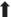	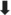
*ALOX15* ‐ Arachidonate 15‐lipoxygenase	1.31	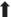	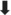
**Polyamine metabolism**			
*SMS* ‐ Spermine synthase	−1.1	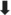	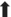
*ARG2* ‐ Arginase 2	−1.14	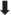	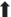
*PAOX* ‐ Polyamine oxidase	−1.05	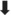	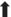
*ODC1* ‐ Ornithine decarboxylase 1	1.07	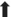	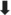

Abbreviations: ADT, androgen deprivation therapy; BMI, body mass index; DEGs, differentially expressed genes; PSA, prostate‐specific antigen; RP, radical prostatectomy.

The most significant GO processes included various processes related to regulation of ion transport, multicellular and anatomical development, digestive system process, and regulation of trans‐synaptic signaling (Figure 3C).

## 4 DISCUSSION

Reports widely describe the relevance of molecular subtyping of PCa and the T2E translocation as a different subtype for localized PCa tumors, although it has different frequencies across populations/ethnicities.^7^, ^26^ However, the contradictory evidence for these subtypes with prognosis remains. In this study, we compared gene expression between *ERG*_*high*_ and *ERG*_*low*_ tumors and found 150 DEGs with an FDR <0.01 and FC >2 that differentiated both groups and interestingly DEGs clustered most of the non‐BCR cases (60%) into the ERG_*high*_ group and most of the BCR cases (60.8%) in the *ERG*_*low*_ group, through unsupervised clustering hierarchical analysis. In accordance, the clinicopathological analysis revealed that more than 60% of the BCR cases occurred in the *ERG*_*low*_ group, and the survival analysis showed a correlation between the *ERG*_*low*_ group with a lower BCR‐free survival, while *ERG*_*high*_ tumors exhibited a better prognosis.

This correlation has been previously reported, showing a BCR‐free survival significantly longer in patients with *ERG* overexpression,^8^ and *ERG* negative status associated with poorer BCR‐free survival in Caucasians, although no association was found for African Americans.^7^ Also, low expression levels of *ERG* have been proposed as an independent predictor for BCR in low‐risk patients.^9^ Moreover, a very recent report also found that negative *ERG* expression measured by immunohistochemistry was associated with biochemical progression after RP.^27^ Our findings may indicate that measurement of *ERG* expression could also be used as a predictor of disease progression in patients treated with RP, including admixed populations such as are Hispanic/Latino population.

However, some studies have also reported opposite results, with *ERG* expression associated with unfavorable outcomes^10^, ^11^ or no correlation with the progression of the disease.^28^, ^29^, ^30^ These dissimilarities across studies may be due to the sampling of the tissues studied, in which only sections of tissue cores,^28^ biopsies,^29^ or frozen tumors^31^ were used, and therefore multifocality and heterogeneity might be underrepresented.

Another important finding in our clinicopathological analyses was the significant differences in Gleason Grade at RP between *ERG*_*high*_ and *ERG*_*low*_ groups (*p* = 0.036), with *ERG*_*low*_ group having higher frequency of higher Gleason Grades and *ERG*_*high*_ group enriched in lower Gleason Grades. Although Gleason Grades G1 (n = 22) and G2 (n = 24) were more frequent in our population with a total of 59% of the patients, the distribution of cases in the *ERG* groups was an interesting finding in our study. This result is consistent with previous evidence showing a correlation between the overexpression of *ERG* or presence of the T2:E translocation with a favorable pathology of lower Gleason scores (≤7), lower primary Gleason pattern (≤ grade 3) and lower Clinical T‐stage (T1 + T2).^32^, ^33^ Hence, our findings between Gleason Grades and *ERG* groups suggest that *ERG*_*low*_ cases may be associated with more advance stages of the disease while *ERG*_*high*_ cases with lower stages.

We also found through univariate analysis that Gleason GG2 at RP was associated with risk of BCR (OR = 3.73; 95%CI 1.12–13.69; *p* = 0.037), and for GG4/5, we observed the same risk direction, although it was not statistically significant (OR = 4; 95%CI 0.86–21.05; *p* = 0.084). This result is consistent with previous evidence showing the prognostic value of Gleason for BCR.^34^, ^35^, ^36^ Nevertheless, in our multivariate analysis including the *ERG* status, the association of Gleason Grade with BCR was lost, which may reflect the influence of other factors not considered in this paper involved in the development of BCR. However, we cannot omit the limitation in our sample size, discussed ahead in the limitations section.

Since it has been previously shown that a higher incidence, mortality, and aggressive presentation of PCa is associated with African ancestry compared with other ethnic groups,^37^, ^38^, ^39^ we wanted to determine whether genetic ancestry in Colombian patients plays a role in the aggressiveness of PCa. Only Indigenous and European ancestries were tested since they sum for the major genetic component in the population included in this study, but a modification of DEGs was not seen. African ancestry was not included given that the median percentage in our cases accounted for a 5%, representing an extremely low component in most of them. Our results suggest that Indigenous and European genetic ancestry have no influence in differential expression profiles between *ERG*_*high*_ and *ERG*_*low*_ cases, while for African ancestry, the data were insufficient to draw conclusions. A more representative population of this ancestry is needed.

To further understand the molecular drivers related to the progression of PCa tumors, we submitted the 150 DEGs to enrichment analysis in MetaCore. It identified signaling pathways related to the angiotensin system, the Protein folding and maturation_Angiotensin system maturation and the renin‐angiotensin system (RAS). RAS is well known for its role in maintaining cardiovascular homeostasis, electrolyte balance, renal physiology, blood pressure, and cell survival.^40^ However, the role of the dysregulated pathway in tumors and the effects on cancer of inhibitors of RAS (RASi) is still unclear.^41^, ^42^

In normal prostate tissue, RAS signaling contributes to spermiogenesis, sperm motility, and survival.^42^ However, different studies imply a dysregulated expression of RAS signaling associated with increased risk of PCa and progression, such as the case of Angiotensin II affecting cell morphology, proliferation, and survival of normal prostate cells through increasing metalloproteinases and regulation of *BAX* and *BCL2*.^43^ *BCL2* contributes to the release and infiltration of *CCL2* protein, which accelerates cancer progression and correlates with high PSA.^44^ Angiotensin II also triggers the *IGFR1*/*AKT* pathway in androgen‐dependent PCa cells transforming them into androgen‐resistant.^45^ Another member of the RAS pathway, the angiotensin II receptor type 1 (*AGTR1*) was also associated with metastatic PCa cells,^46^ while the angiotensin II receptor type 2 inhibits tumor growth, induces apoptosis, and reduces Ki‐67 and AR expression.^47^ Nevertheless, expression of RAS components has been identified in prostate tissues, and especially highly expressed in resistant PCa cases compared with untreated and normal prostate tissue.^48^

RASi are widely used for the treatment of cardiovascular and renal diseases in the form of angiotensin receptor blockers (ARB) or angiotensin‐converting enzyme inhibitors (ACEIs).^50^ Studies evaluating the impact of RASi on PCa show consistent and favorable results. Among hypertensive patients, long‐term use of ARBs or ACEI reduced the risk of PCa,^51^ ARB‐treated veterans showed a small but significant reduction in the incidence of PCa,^52^ and the intake of ACEIs/ARBs associated with a significantly reduced risk of BCR after radiotherapy with adjuvant/neoadjuvant hormone treatment.^53^ Given the growing evidence that RASi have a role in reducing the risk and progression of PCa, and that we identified angiotensin related signaling as enriched pathways in *ERG*_*high*_ tumors, which were associated with a reduced risk of BCR compared with the *ERG*_*low*_ tumor group, together these results warrant further research exploring RASi in patients with T2E arrangements or differential expression of *ERG* and different subtypes. Nonetheless, the RAS system appears to be implicated in PCa tumors for which RASi could improve patient management and outcomes.

Another enriched pathway identified through MetaCore and related to metabolism was the linoleic acid metabolism signaling pathway, with members of this pathway downregulated (*CYP2J2*, *ALOX15B*, *FAD52*, [Table 3]) in the *ERG*_*high*_ group and overexpressed in *ERG*_*low*_ tumors. The involvement of linoleic acid in cancer and in general in cardiovascular health is still unclear. Considered as an essential omega‐6 fatty acid, the consumption of linoleic acid was markedly increased in the past century given by the dietary recommendations in the U.S and Western countries.^53^ Then, a relationship between dietary linoleic acid consumption and the development of some cancers was suggested, however, the findings are contradictory.^54^, ^55^, ^56^ For PCa, some of the reports show no association of individual n‐6 fatty acids with this type of cancer,^57^, ^58^ but a trend for n‐3 fatty acids as a protective factor was reported for Latinos and Whites (compared with African Americans and Japanese Americans).^58^ A higher ratio of n‐6/n‐3 fatty acids intake, however, was associated with an increased risk of high‐grade PCa.^59^ Another study reported that intakes of saturated fats were related to the risk of advanced or fatal PCa, but no association between total n‐6 or ratio n‐6/n‐3 fatty acids and risk of PCa was found.^60^ Recently, Figiel et al.^61^ indicated that low levels of linoleic acid and high levels of saturates characterized the profile associated with PCa aggressiveness in African‐Caribbeans, which was analyzed in the periprostatic adipose tissue of patients. In contrast, animal and in vitro experiments have had more consistent findings, suggesting that n‐6 fatty acids stimulate PCa growth, whereas n‐3 inhibits it.^62^, ^63^ Meller et al.^64^ discovered that tumors with the T2E translocation have a different metabolome profile compared with the T2E negative tumors, particularly enriched in fatty acids and suggesting that the metabolism of fatty acids in PCa tumors could be modulated depending on the presence of the translocation; it was found however that linoleic acid was increased in T2E positive tumors. Considering these findings, studies exploring the role of linoleic acid metabolism in a T2E translocation context should be done.

Finally, the polyamine metabolism pathway is frequently dysregulated in cancer given the need for polyamines for transformation and tumor progression.^65^ The polyamines include putrescine, spermidine, and spermine, which are polycations involved in cell growth, survival, protein and nucleic acid synthesis, stabilization of chromatin structure, differentiation, apoptosis, nucleic acid depurination, and major components of prostate fluid.^66^ Different known oncogenic pathways lead to the dysregulation of polyamine metabolism, including *MYC* signaling, *RAS*/*RAF*/*MEK*/*ERK* signaling pathway, *AKT* signaling^66^ *PTEN*/*PI3K*/*mTORC1*,^67^ and the activation of the non‐canonical *WNT* signaling pathway which appears to be associated with decreased citrate and spermine levels in the most aggressive phenotypes of PCa.^68^ In line with this evidence, Meller et al.^64^ reported that putrescine and spermine were decreased in prostate cancer compared to normal tissues, while spermidine was increased, and under the context of T2E translocation, a negative correlation of spermine and putrescine with *ERG* rearrangement was described.^18^, ^64^ In our study, we observed downregulation of some members of the polyamine metabolism pathway in *ERG*_*high*_ tumors, but we cannot infer which polyamines are affected. Thus, this data indicates that alteration of polyamine metabolism is involved in prostate tumors, however, their participation in tumor progression under the context of *ERG* rearrangements should be addressed. Finally, polyamines and polyamine metabolites measured in either urine or serum have shown potential as biomarkers for prostate cancer,^69^ which also could assist in tumor subtyping and personalized medicine.

Since this is a Hospital‐based retrospective study, there are some limitations that are important to discuss and that could be explained in part by our institutional context, that is, a high number of the patients treated at the INC experience many barriers to health access during their treatment and follow‐up, including transportation and a deeply fragmented and segmented health system that affects patients every time their health insurer decides to change the cancer institute for their management, causing delays in their clinical interventions. The retrospective design and short follow‐up restricted the analysis for other survival events. The small sample size and the inclusion of only PCa patients with localized/regionally advanced PCa that underwent RP surgery, may explain the distribution in the frequencies of low and high Gleason Grades and the strength of associations. Given the nature of a retrospective study with follow‐up information, FFPE tissues were used. To overcome quality issues, we used optimal and suited laboratory protocols for nucleic acids extraction, combined with feasible protocols for subsequent NGS‐based analysis of these molecules.^71^ A a bias in the African genetic ancestry representation for analysis might not allow conclusive results given that patients from the INC were mainly from the Andean region and few from the Coastal region, which have higher African ancestry in our country. Finally, incident metastatic PCa cases were ruled out since they were inoperable, therefore, aggressive cases were underrepresented in our study.

Overall, our study confirmed a differential BCR‐free survival for *ERG* tumor groups, defined as *ERG*_*high*_ and *ERG*_*low*_, in Hispanic/Latino patients with localized/regionally advanced PCa, with *ERG*_*low*_ tumor cases having the worst survival. Analysis of enriched pathways in these groups found metabolism‐related pathways, such as the renin‐angiotensin system, the linoleic acid metabolism, and polyamines metabolism. Since the pathways we found are altered in *ERG*_*high*_ and *ERG*_*low*_ tumors, and *ERG*_*low*_ tumors were correlated with a shorter BCR‐free survival, the results may suggest an involvement of these pathways in BCR. Although these findings should be confirmed in larger and more diverse populations in Hispanics, it warrants further research concerning diet, metabolism and lifestyle factors into prevention and management of this type of cancer, since these are considered as modifiable risk factors, as well as to better understand the interaction between the *ERG* status with Gleason Grades and the prognosis of PCa.

## AUTHOR CONTRIBUTIONS

**Natalia L. Acosta‐Vega:** Conceptualization (equal); data curation (lead); formal analysis (lead); investigation (lead); methodology (equal); writing – original draft (lead). **Rodolfo Varela:** Conceptualization (equal); data curation (equal); supervision (supporting); writing – review and editing (equal). **Jorge Andrés Mesa:** Conceptualization (equal); data curation (equal); supervision (supporting); writing – review and editing (equal). **Jone Garai:** Data curation (equal); methodology (equal); writing – review and editing (equal). **Melody C Baddoo:** Data curation (equal); formal analysis (equal); methodology (equal); software (equal); writing – review and editing (equal). **Alberto Gómez‐Gutiérrez:** Conceptualization (equal); data curation (equal); formal analysis (equal); methodology (supporting); resources (supporting); supervision (lead); writing – review and editing (equal). **Silvia J. Serrano‐Gómez:** Formal analysis (equal); methodology (equal); writing – review and editing (equal). **Marcela Nuñez Lemus:** Methodology (supporting); writing – review and editing (supporting). **Martha Lucía Serrano:** Formal analysis (supporting); writing – review and editing (supporting). **Jovanny Zabaleta:** Conceptualization (equal); data curation (equal); formal analysis (equal); funding acquisition (equal); methodology (equal); resources (equal); supervision (supporting); writing – review and editing (equal). **Alba Lucia Combita:** Conceptualization (equal); data curation (equal); formal analysis (equal); funding acquisition (equal); methodology (equal); project administration (equal); resources (equal); supervision (lead); writing – review and editing (equal). **María Carolina Sanabria‐Salas:** Conceptualization (equal); data curation (equal); formal analysis (equal); funding acquisition (equal); methodology (equal); project administration (equal); resources (equal); supervision (lead); writing – review and editing (equal).

## FUNDING INFORMATION

Instituto Nacional de Cancerología (MCSS project funding C41030310118 and ALC project funding C19010300456). Translational Genomics Core Laboratory at LSUHSC‐New Orleans ‐ USA (JZ grants: P30GM114732, P20GM121288–01, P20CA202922).

## CONFLICT OF INTEREST

The authors declare no potential conflicts of interest.

## ETHICS STATEMENT

This study was approved by the Research Ethics Board of the Colombian National Cancer Institute and was designated as an exempt study for informed consent.

## Supporting information


Figure S1
Table S1

## Data Availability

The data generated in this manuscript is deposited in NCBI’s Gene Expression Omnibus (GEO) through the accession number GSE216490. Expression profile data to confirm the association between ERG expression with BCR was obtained from the Gene Expression Omnibus (GEO) at GSE70770.
