# Supplementary material for: Metabolic pathways enriched according to ERG status are associated with biochemical recurrence in Hispanic/Latino patients with prostate cancer
Source: Cancer Med. 2022 Nov 3;12(4):4306–20. doi: 10.1002/cam4.5301 (PMC9972164; doi:10.1002/cam4.5301)
Supplement: Supplementary file 1 — Figure S1 Table S1 [file CAM4-12-4306-s001.docx]

***Metabolic pathways enriched according to ERG status are associated with biochemical recurrence in Hispanic/Latino patients with prostate cancer***

**Supplementary material.**

**Supplementary Figure 1. Heatmap for the 532 DEGs.** Unsupervised hierarchical clustering analysis for 78 PCa tumors samples from PCa patients. DEGs were obtained from a comparison between *ERG_high_* and *ERG_low_* groups (FDR<0.01). Normalized counts of expression were scaled, and expression values for each gene were color labeled (blue to red). Patients are represented in columns and genes in rows.


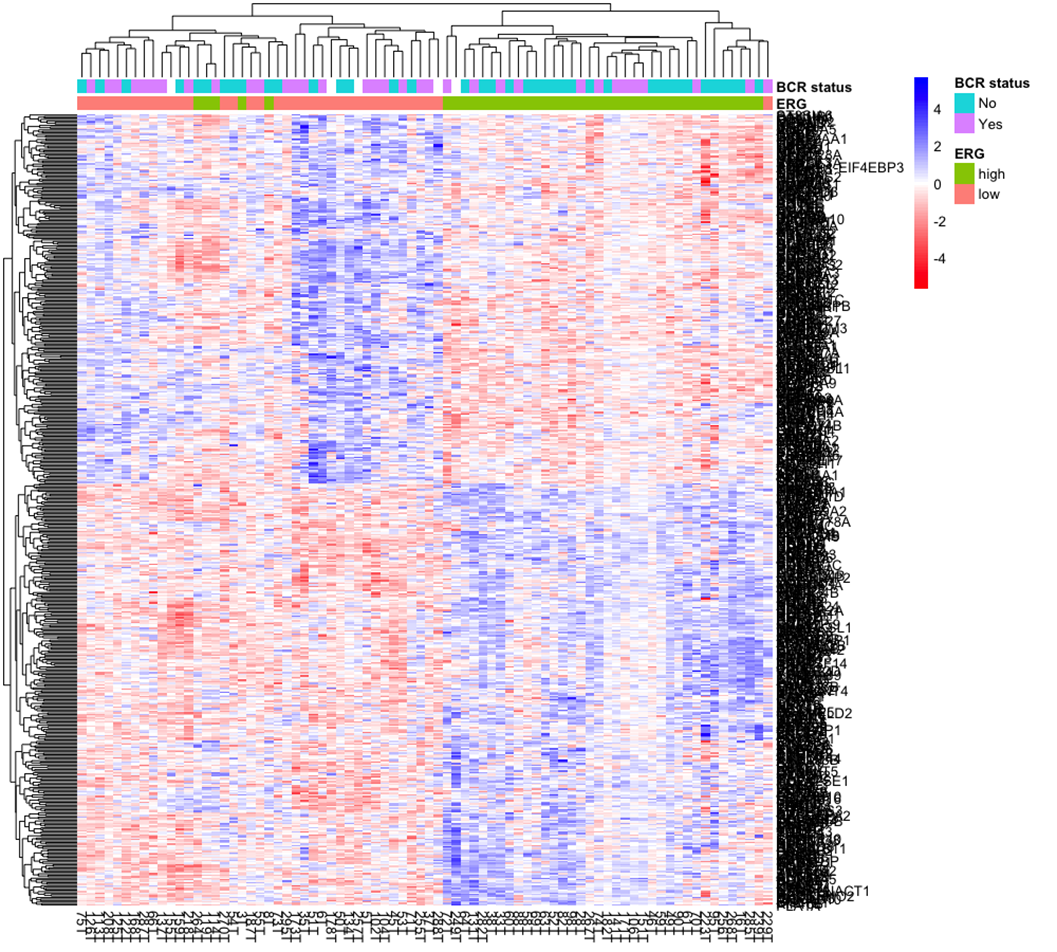


**Supplementary Table 1.** Distribution of clinical and pathological characteristics by clusters of *ERG*.

|  | *ERG_low_* | *Cluster 2-ERG_high_* | *ERG_high_* | *P* |
| --- | --- | --- | --- | --- |
| n | **26** | **15** | **37** |  |
| Age - years (median, range) | 66.00 (32.00, 73.00) | 65.00 (48.00, 73.00) | 64.00 (42.00, 73.00) | 0.712 |
| BMI (median, range) | 27.04 (17.28, 35.19) | 25.34 (20.45, 30.12) | 26.01 (17.71, 36.00) | 0.311 |
| Ancestry |  |  |  |  |
| European ancestry (median, range) | 0.58 (0.26, 0.81) | 0.46 (0.19, 0.66) | 0.58 (0.35, 0.78) | 0.137 |
| Indigenous ancestry (median, range) | 0.36 (0.05, 0.66) | 0.41 (0.14, 0.53) | 0.37 (0.01, 0.65) | 0.515 |
| African ancestry (median, range) | 0.06 (0.00, 0.56) | 0.06 (0.00, 0.58) | 0.04 (0.00, 0.40) | 0.444 |
| Pre-operative characteristic |  |  |  |  |
| Preoperative PSA (median, range) | 9.87 (4.40, 45.21) | 8.20 (2.94, 44.00) | 9.41 (3.70, 31.97) | 0.83 |
| Clinical stage (%) |  |  |  |  |
| I | 8 (30.8) | 3 (20.0) | 13 (35.1) | 0.598 |
| II | 17 (65.4) | 12 (80.0) | 24 (64.9) |  |
| IV | 1 (3.8) | 0 (0.0) | 0 (0.0) |  |
| Gleason GG at biopsy (%) |  |  |  |  |
| GG1 | 8 (36.4) | 8 (57.1) | 25 (69.4) | 0.273 |
| GG2 | 6 (27.3) | 3 (21.4) | 6 (16.7) |  |
| GG3 | 6 (27.3) | 2 (14.3) | 3 (8.3) |  |
| GG4 and GG5 | 2 (9.1) | 1 (7.1) | 2 (5.6) |  |
| D'Amico risk groups (%) |  |  |  |  |
| LR | 7 (26.9) | 4 (26.7) | 10 (27.0) | 1 |
| IR | 12 (46.2) | 7 (46.7) | 17 (45.9) |  |
| HR | 7 (26.9) | 4 (26.7) | 10 (27.0) |  |
| Post-operative characteristic |  |  |  |  |
| % tumor in RP (median, range) | 23.00 (1.00, 90.00) | 20.00 (1.00, 75.00) | 17.00 (2.00, 60.00) | 0.652 |
| Gleason Grade Group at PR (%) |  |  |  |  |
| G1 | 3 (11.5) | 3 (20.0) | 16 (43.2) | **0.037** |
| G2 | 11 (42.3) | 2 (13.3) | 11 (29.7) |  |
| G3 | 7 (26.9) | 5 (33.3) | 6 (16.2) |  |
| G4 and G5 | 5 (19.2) | 5 (33.3) | 4 (10.8) |  |
| Pathological stage (%) |  |  |  |  |
| T1/T2 | 14 (53.8) | 5 (33.3) | 22 (59.5) | 0.242 |
| T3 | 12 (46.2) | 10 (66.7) | 15 (40.5) |  |
| Extracapsular extension in RP (%) |  |  |  |  |
| No | 14 (53.8) | 5 (35.7) | 19 (52.8) | 0.54 |
| Yes | 12 (46.2) | 9 (64.3) | 17 (47.2) |  |
| Lymph node compromise (%) |  |  |  |  |
| No | 24 (92.3) | 10 (66.7) | 33 (89.2) | 0.078 |
| Yes | 2 (7.7) | 5 (33.3) | 4 (10.8) |  |
| BCR (%) |  |  |  |  |
| No | 9 (39.1) | 8 (57.1) | 22 (61.1) | 0.249 |
| Yes | 14 (60.9) | 6 (42.9) | 14 (38.9) |  |
| ERG (%) |  |  |  |  |
| low | 26 (100.0) | 9 (60.0) | 2 (5.4) | **<0.001** |
| high | 0 (0.0) | 6 (40.0) | 35 (94.6) |  |

Abbreviations: BMI, body mass index; PSA, prostate-specific antigen; RP, radical prostatectomy.
